# Modeling Grid Cell Distortions with a Grid Cell Calibration Mechanism

**DOI:** 10.34133/cbsystems.0140

**Published:** 2024-09-12

**Authors:** Daniel Strauß, Zhenshan Bing, Genghang Zhuang, Kai Huang, Alois Knoll

**Affiliations:** ^1^ Chair of Robotics, Artificial Intelligence and Real-time Systems, TUM School of Computation, Information and Technology, Technical University of Munich, Munich, Germany.; ^2^ School of Data and Computer Science, Sun Yat-sen University, Guangzhou, China.

## Abstract

The medial entorhinal cortex of rodents is known to contain grid cells that exhibit precise periodic firing patterns based on the animal’s position, resulting in a distinct hexagonal pattern in space. These cells have been extensively studied due to their potential to unveil the navigational computations that occur within the mammalian brain and interesting phenomena such as so-called grid cell distortions have been observed. Previous neuronal models of grid cells assumed their firing fields were independent of environmental boundaries. However, more recent research has revealed that the grid pattern is, in fact, dependent on the environment’s boundaries. When rodents are placed in nonsquare cages, the hexagonal pattern tends to become disrupted and adopts different shapes. We believe that these grid cell distortions can provide insights into the underlying neural circuitry involved in grid cell firing. To this end, a calibration circuit for grid cells is proposed. Our simulations demonstrate that this circuit is capable of reproducing grid distortions observed in several previous studies. Our model also reproduces distortions in place cells and incorporates experimentally observed distortions of speed cells, which present further opportunities for exploration. It generates several experimentally testable predictions, including an alternative behavioral description of boundary vector cells that predicts behaviors in nonsquare environments different from the current model of boundary vector cells. In summary, our study proposes a calibration circuit that reproduces observed grid distortions and generates experimentally testable predictions, aiming to provide insights into the neural mechanisms governing spatial computations in mammals.

## Introduction

Within the mammalian brain, there exists a biological system that possesses the potential to improve our understanding of neural circuitry and provide substantial insights into navigational computation. This system is linked to the processes of navigation and self-location and has been subject to extensive research efforts, which have yielded several noteworthy findings. For instance, a growing set of functional cell types that encode spatial information is currently being discovered. Through the analysis of these cell types, researchers have gained valuable insights into the possible underlying computations and have developed a multitude of theoretical models. Although there exist other neural systems whose neuronal behavior can be described with similar precision, most of them are computationally situated at a proximate stage to the sensory input or muscular output [1]. This particular system is situated farther from the sensory inputs and motor outputs, which is a property that may reveal distinct principles of neuronal computation that systems closer to input and output cannot reveal.

The initial discovery pointing to the existence of this system dates back 50 years when neurons with unique firing properties in the hippocampus were discovered. These neurons, known as place cells (PCs), fired every time a freely moving rat visited a particular location in the cage [2] (see Section “Functional cell types of navigation in the hippocampal formation”) (it should be noted, however, that spatial representations are not limited to the hippocampal formation, as other regions displaying similar properties have been discovered more in later studies [3,4]). This was a discovery for which John O’Keefe was awarded one-half of the Nobel Prize in Physiology or Medicine. The other half was given to May-Britt Moser and Edvard I. Moser for the discovery of another neuronal cell type [5], which provided important insights into the workings of this system. Specifically, by plotting the firing positions of one of these cells, the resulting points formed a hexagonal grid, a remarkable finding. These cells, called grid cells (GCs) (see Section “Functional cell types of navigation in the hippocampal formation”) are located in the medial entorhinal cortex (mEC) [6], a region closely connected to the hippocampus. PCs and GCs encode very similar types of spatial information, suggesting that they are part of the same navigation system. Subsequent studies have led to the discovery of many other cell types in these regions that encode information about position, providing further evidence for the existence of a navigation system in this part of the brain [7–10].

The phenomenon of so-called “grid distortions” or “GC distortions” in GCs is a puzzling and interesting finding, wherein the symmetry of the periodical firing pattern tends to be distorted when rodents are placed in a differently shaped cage. The resulting firing pattern is no longer a point-symmetric hexagonal pattern. Depending on the shape of the new cage, some of the observed firing peak transformations can be approximated by a linear transformation, while others have to be approximated with a nonlinear transformation [11–13].

**Fig. 1.**
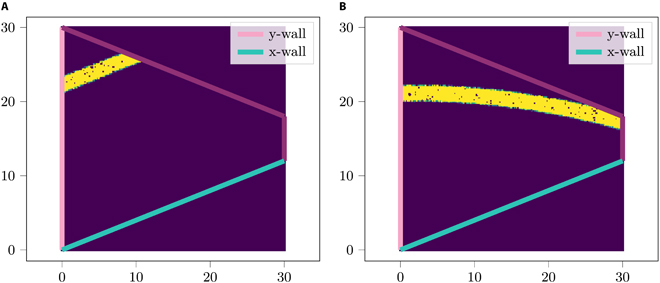
(A) Firing field of a BVC based on the current BVC model encoding distance to x-wall. (B) One potential firing field of a BVC encoding distance to x-wall according to an alternative BVC behavior proposed in this study.

This work aims to provide a possible explanation for grid distortions on a neuronal level by extending an earlier GC model with a calibration circuit based on the biological findings of this system. The new model’s behavior should resemble experimentally found grid distortions to validate it. Additionally, the model incorporates the well-established findings of the influence of environmental boundaries on PCs [13,14]. This work aims to develop a biologically plausible description of the system, taking us one step closer to understanding this phenomenon. The contributions of this work are summarized as follows:

• A comprehensive model of GCs, PCs, boundary vector cells (BVCs), and border cells (BCs) is presented that incorporates a calibration system for GCs to reproduce grid distortions as a byproduct.

• To the best of our knowledge, this model is the first to replicate not only grid distortions but also other observed phenomena related to PCs and speed cells.

• It is typically difficult to find strong evidence on questions regarding the relationship between different functional cell types. Other publications have proposed different hypotheses and ideas regarding these connectivities [12,15–17]. Our model provides support for 2 of these ideas:

– GCs receive functional input from PCs.

– PCs receive essential input from BVCs and BCs.

• Our model also makes 2 experimentally testable predictions:

– An alternative model for BVCs is suggested in which a proportion of these cells encode relative distance rather than absolute distance. This prediction can be tested by examining the behavior of BVCs inside a trapezoid; if along the boundary encoded by a BVC the thickness of the cage changes, then the BVC’s field might not be entirely parallel to this wall but curved toward it; Fig. 1 illustrates this prediction.

– The model makes a prediction of the shape of grid fields inside a square cage, if rats did grow up inside a nonrectangular trapezoidal cage.

### Biological background

#### Functional cell types of navigation in the hippocampal formation

Functional cell types are defined by the behavior of their constituent cells. This section discusses several functional cell types within the hippocampal formation that contribute to spatial navigation, providing a foundational idea of this system.

PCs are a prominent neuronal type of typically situated in the hippocampus. A PC fires each time the animal visits one particular location. Consequently, by knowing the firing peak location of each PC of an animal, one can approximate the animal’s current location by solely examining the current firing activity of the PCs, without needing to observe the animal itself [2].

GCs [6] are located in the mEC. These cells can be identified by monitoring their firing activity as the animal traverses a cage and marking each location where the cell fires. If the firing locations form a hexagonal grid, the cell is classified as a GC. While there is evidence that GCs are involved in the computation of path integration, the meaningfulness of the current experimental evidence is being questioned, so that this idea is considered only a possibility [15,18–20]. The GC model used in this work also performs path integration (see Section “GC model”), but it can be adapted with minimal changes to produce the same firing behavior without GCs performing path integration.

BCs are located in the mEC and are known to fire when an animal is in close proximity to an environmental boundary, with the boundary located at a specific allocentric direction from the animal [9,10]. There exist theories and hypotheses that BCs participate in calibrating GCs [21], as we will further discuss in Section “Connectivity of these cells”.

BVCs are located in the subiculum and fire maximally when a boundary is at a specific allocentric direction and distance from the animal [8]. BVCs differ from BCs in that they only fire when the animal is in close proximity to a border, so functionally BCs can be seen as a special case of BVCs. The role of BVCs in providing input to PCs has been a topic of discussion, which will be further addressed in Section “Connectivity of these cells”.

It is worth noting that certain cells display firing patterns that qualify them for multiple types of cells described above [22]. Therefore, the behavior of numerous cells cannot always be definitively categorized into one of the aforementioned types.

#### Connectivity of these cells

To understand how the underlying circuits work, it is insufficient to know which functional cell types are involved. One also has to know how these cells interact, which cell provides functional input for which cell, and how cells that can be categorized as none of these cell types are involved in this system.

The Grid- and PC Relationship. Several studies have been conducted to explore the relationship between GCs and PCs. The review [20] provides an overview of studies, particularly focusing on which of the 2 cell types provides functional input to the other.

A collection of physiological studies is presented, supporting the notion that GCs contribute to the spatial firing of PCs. Specifically, it has been observed that PCs receive excitatory inputs from GCs along with other spatial and nonspatial cell types [23]. Moreover, it has been observed that PCs exhibit greater precision in the proximal CA1 zone in the hippocampus than in the distal CA1 zone, which coincides with a larger input from the mEC to the proximal CA1 compared to the distal CA1 [24]. Additionally, removing the input from the mEC to the CA1 region has been found to impact PCs, resulting in fewer cells acting like PCs and larger field sizes from the remaining PCs. However, when the input from GCs to PCs was removed, the place fields did not disappear entirely and were still able to function to some extent [25].

Additional evidence supports the idea that GCs are not necessary for PCs to function to some degree. Mature PCs can be seen immediately after rats leave the nest, whereas stable grid fields appear several days later [26,27]. Furthermore, some evidence suggests that GCs receive functional input from PCs, further weakening the idea that PCs are only driven by GCs. The entorhinal cortex receives input from the hippocampus [28,29], and inactivation of the hippocampus resulted in GCs no longer producing a hexagonal grid pattern [30]. These findings provide additional indications that GCs may receive functional input from PCs.

In conclusion, there is evidence to (not necessarily strongly) support both connectivity hypotheses, and it appears that PCs, while assuming they receive functional input from GCs, do not rely solely on GCs. Therefore, it has been hypothesized in [20] that PCs are more strongly driven by extrinsic sensory cues, while GCs play a supportive role. Additionally, based on the evidence presented in this section, it has been suggested that GCs and PCs form a loop in which they support each other in their functions, with GCs also receiving input from PCs [17,20].

Hence, the GCs in this work’s model are calibrated from PCs. For the sake of simplification, we have not incorporated connectivity from GCs to PCs in this study, which can be a future extension.

The Input to PCs. If PCs can function without GCs (as discussed in the previous paragraph), from which other cells do they potentially receive spatial input (possibly as well) to produce their fields?

It has been discovered that PC firing locations are, to some extent, aligned with the environmental surroundings. For example, scaling up the size of the environment results in a proportionate scaling of place fields [14]. Additionally, the movement of an environmental wall results in a corresponding movement of place fields close to the wall, with those PCs tending to stay in proximity to the wall [13]. It has also been observed that PCs tend to have the same position relative to an environmental border [31].

Some experiments also suggest the possibility of inputs to PCs from cells other than BVCs, BCs, or GCs. Specifically, some hippocampal cells have been found to encode the location of cues so that they acted like PCs [32,33]. When the cues were moved, the cells’ firing moved with the cues; when the cues were removed, the cells still fired at the last location. This leads to the hypothesis that object vector cells might also provide input to PCs. It appears that other environmental sensory inputs, beyond those encoding spatial relations to the boundaries, could also impact PCs.

The Grid- and BVC Relationship. Are there direct connections between BVCs and GCs without intermediary neurons? Some evidence suggests that GCs may receive functional inputs from cells that encode information about environmental boundaries, such as BVCs and BCs do. Studies are pointing out the dependence of the grid field on environmental borders [34,35]. Also, studies discussed in Section “GC and PC distortions” have demonstrated distortions in grid patterns resulting from manipulations of environmental boundaries. Additionally, the study in [21] has shown that the error of GC firing accumulates over time and distance since the last boundary encounter. These findings provide suggestive evidence for the hypothesis that GCs may functionally receive information carried on by BVCs and BCs. If the hypothesis of calibration inputs from BVCs and BCs to GCs holds true, it raises the question of how the underlying circuitry might be organized.

How plausible is the assumption of direct connections from BVCs to GCs without the involvement of intermediary neurons? If such connections exist and BVCs can indeed provide corrective input to GCs, it follows that a linear combination of the firing fields of BVCs should exist that bears some resemblance to the firing field of a GC. Investigating the existence of such a linear combination through simulations or analytical analysis would offer insights into the viability of the direct connection between GCs and BVCs. In the present study, we investigate the converse scenario, wherein intermediary neurons exist within the circuitry linking BVCs and GCs.

Therefore, this avenue poses a possibility for future work.

What would be the most straightforward approach to modeling such a connection? Let us consider the hypothetical scenario where constructing a neural circuit that functions similarly to a logical AND gate is biologically feasible. This circuit would consist of 2 input neurons, *i*_1_ and *i*_2_, and an output neuron, *o*, such that the firing rate of *o* would gradually reach the minimum firing rate of *i*_1_ and *i*_2_ over time. If an AND-gate circuit has 2 nonparallel BVCs as input cells, the output cell might behave as a PC, provided the environment has the appropriate shape. In related computational works, that predicted the existence of BVCs, PCs also were driven by BVCs [36,37].

#### GC and PC distortions

In the field of GC research, grid distortions refer to the phenomenon whereby the hexagonal grid pattern produced by GCs deviates from regularity. This can occur due to changes in environmental boundaries, as will be elaborated upon later. In the present section, we aim to present some examples of grid distortions observed in experimental studies, which we want to reproduce later.

*Distortions in Nonsquare Rectangular Environments*

One noteworthy grid distortion was discovered in [12] where rats were familiarized with a square cage and the grid recorded in this cage was found to be symmetrical. When the cage was subsequently scaled down along one axis to a rectangular shape, the resulting grid in the new environment was also scaled down along the same axis. Conversely, if the rats were familiarized with a rectangular environment, the grid was found to be symmetrical within that environment. However, if the cage was scaled up to a square environment, the grid was scaled up along the same axis as well. The simulation results replicating this observation are discussed in Section “Grid distortions in nonsquare rectangles”.

*Distortions in Nonrectangular Trapezoidal Environments*

Grid distortions have also been reported in a study involving animals navigating in nonrectangular trapezoidal environments [11]. The trapezoid used in these studies was an isosceles trapezoid, with one half being narrower than the other. Upon examining the autocorrelograms of the GC firing fields, a distinct distortion was observed in the new environment in some GCs. This distortion can be characterized as a shear transformation, as indicated by the increased ellipticity (the impact of the distortion varied among different GC modules) of the grid within the trapezoid. Furthermore, a change in the orientation and wavelength of the grid was observed throughout the trapezoid. According simulation results are discussed in Section “Grid distortions in nonrectangular trapezoids”.

*Environmental Changes Influence Also PCs*

In a study by Krupic et al. 2018 [13], the effect of environmental changes on not only GCs but also PCs was investigated. The experiment consisted of 2 groups where 1 cage wall was gradually expanded or contracted. The original shape of the cage was a nonrectangular polygon with 5 angles. The expansion transformed it into a rectangle in one group, while the contraction turned it back into a polygon in the other group. This allowed for matching the firing peaks of GCs or PCs in the polygon to those in the rectangle. Grid peaks close to that wall, which was moved, moved in the same direction as the wall. Peaks further away from this wall moved by a far smaller distance. Additionally, the peaks of PCs close to the wall were moved in the same direction as the wall, but by a further amount than GCs. From analyzing the correlation of the shift of GCs vs PCs, the suggestion has been made that there might be interactions between some PCs and GCs and that other PCs do not interact with GCs. Due to the nonsignificant correlation between the GC and PC shift in this work, it has also been suggested that GCs are influenced not only by PCs but also by other spatial cells at the same time [13]. Our model involves both PCs and GCs; it can be compared to these findings. Another experimental observation against which this model can be assessed is the work in [14]; This research demonstrated that as the scale of an environment increases, a corresponding proportion of the place fields also scales up. Refer to Reproducing PC distortions for simulations that replicate this behavior of PCs.

### Related work

Krupic et al. [16] developed a descriptive model (here, “descriptive” refers to a model that describes the resulting grid pattern in relation to environmental boundaries, without explicitly modeling the underlying neural circuitry) to analytically describe how grid patterns emerge in dependence on environmental boundaries. To construct this model, a dynamic system was created in which a set of grid peaks moved in time across space due to attractive and repulsive forces between each grid peak. This model could predict several experimental observations, including grid alignment to environmental boundaries, distortions in grid patterns caused by changes in environmental geometry, and the phenomenon that grid modules of different sizes produce different distortions. From the way the attractive and repulsive forces were built up in their model, a model about the connectivity of PCs, BCs, and GCs was built. Similar to the work of Krupic et al. in this work, PCs do also provide input for GCs, and PCs also receive input from BCs.

In the work of Monsalve-Mercado and Leibold [15], a model on a neuronal level was constructed that replicates grid distortions. In this model, GCs received inputs from simulated hippocampal cells that contained spatially rich information. The weights are trained via Section “Hebbian learning” and some additional learning rules. With this setup and the additional learning rules, the GCs would develop a grid pattern by themselves, without any artificial setting of weights like in most continuous attractor network (CAN) models for GCs. The model reproduced many experimental findings, including various grid distortions and the tendency of GCs to cluster into one of 3 orientations. In this model, their version of PCs also project to the GCs, in the same way as it has been done in [16] and in this work.

## Materials and Methods

### Experimental design

In this study, we construct a calibration circuit for GCs. We simulate in silico the neurons that make up this circuit and the environmental inputs. The circuit is based on the idea that in mammals, the corrective mechanisms for GCs are, to a sufficiently large degree, driven by the distances from the animal to the environmental boundaries. GCs receive functional input from PCs, and PCs receive functional inputs from BCs and BVCs.

**Fig. 2.**
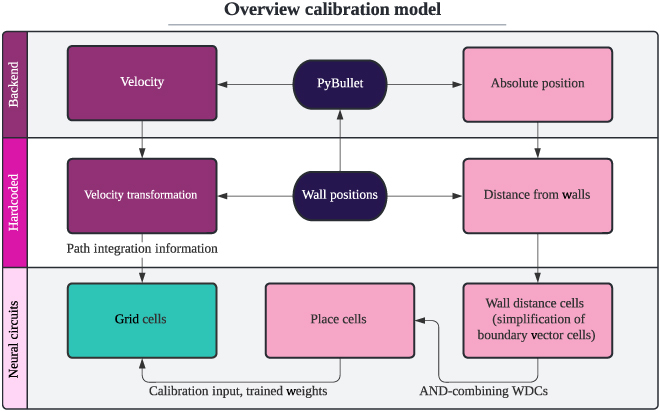
This figure shows an overview of the calibration model. It should be noted that this plan does not present the actual connectivity of these cell types to the best of our knowledge but is a simplification of that for the purpose of building the model. The purple nodes show the input path to the GC network needed for path integration. Velocity transformation might be implemented as a neural circuit in future work. The pink nodes show the calibration input path to GCs, where WDCs simplify BVCs and BCs. The dark purple nodes show the environment and the green node the GCs.

In this model, GCs receive inputs from a PC model (see Section “AND map (PCs)”), and the corresponding weights are trained using a Section “Hebbian learning”implementation (see Section “Hebbian learning”). Additionally, the behavior of the PCs is controlled by wall distance cells (WDCs) (see Section “Wall distance cells”). The construction of the neural circuit responsible for this interaction is described in Section “AND map (PCs)”. Figure 2 shows this model’s components and their relationships.

As explained in Section “Wall distance cells”, WDCs simplify BVCs and BCs, and they encode the distance to given cage walls. We train the calibration mechanism inside a square cage, which calibrates the GCs from WDCs. As the calibration mechanism is trained inside a square cage, we do not expect to observe any grid distortions within it (more information about training and the calibration circuit will be discussed in subsequent sections). We then place the square-trained agent into a nonsquare environment and observe how the calibration output affects the shape of the grid. The nonsquare environment or network parameters will be adapted depending on the specific experiment. We expect to observe grid distortions like those observed experimentally and discussed in Section “GC and PC distortions”. The same procedure as in [38] is used to provide the input data. Specifically, the neuronal model receives movement and position data from a PyBullet simulation in which a virtual P3DX robot, whose model is given by [39], traverses a cage. That way, the PyBullet environment provides velocity data and the distances to the cage walls for our neural model.

### Neuronal model

Unless otherwise stated, we will use the same neuronal model used in the GC model proposed in [40], and the implementation of [41] for a given subnetwork of neurons 0, …, *n* − 1. Let *w* ∈ *R*^*n*×*n*^ be the weight matrix, such that *w_ij_* represents the strength of the connection from neuron *i* to *j* and let *s* ∈ *R^n^* be the spiking vector, where *s_i_* denotes the current firing strength of neuron *i*. During the simulation, we use the following rule to update the spiking vector of the subnetwork:

$$\tau \frac{ds}{dt}+s=\text{max}(w\cdot s+B+1,0)$$ (1)

Vector *B* specifies the subnetworks input; it could originate from another subnetwork or be sensory input; for example, in our GC network, each element in *B* resembles the sum of velocity and calibration input stimulating a given GC. The symbol *τ* denotes the time constant specifying rate of convergence. In this model, *τ* was set to 100 ms.

### GC model

**Fig. 3.**
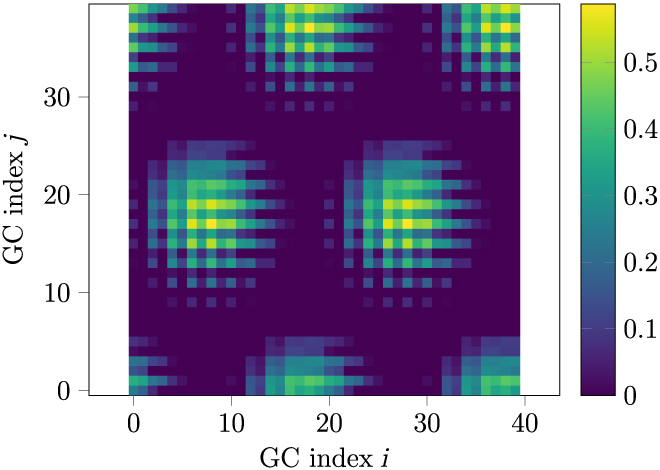
The presented figure provides a visualization of a grid module’s activity at a point in time. Each pixel within the plot corresponds to an individual GC, with its color indicating the current firing strength. This plot arranges GCs by their *i* and *j* indexes. The specific mapping between these indexes and the corresponding GCs is detailed in [41], ensuring that for each pair of GCs, the index offset of the pair is linear to their firing peak offset in the spatial domain.

For our model, we employ the GC model from [42], which is based on the model introduced in [40] and is a CAN. The GC implementation that we use for this model was made in [41], which we slightly modified in order to make the initial grid orientation deterministic. This model consists of an *n* × *n* grid module, whose firing activity at a point in time *t* is presented in Fig. 3.

### Wall distance cells

As the calibration process is dependent on the distances to the environmental borders, it is necessary to establish a neural encoding of these distances. Candidates for cell types encoding this information are BVCs and BCs. These cells fire if an environmental border is at a given distance at a given allocentric angle relative to the animal’s position. For our model, a simplified variant of these cells will be employed, which we will call WDCs. A given WDC fires when the agent is at a given distance from a given cage wall. In order to implement the WDCs, we introduce 2 vectors of neurons: the “*x-vector*” and the “*y-vector*”. The *x-vector* encodes the distance to the cage wall, which was labeled “y-wall” *w_y_*, while the *y-vector* represents the distance to the cage wall that was labeled “x-wall” *w_x_*. Each vector consists of *n* neurons, denoted as *x*_1_, …, *x_n_* for the *x-vector* and *y*_1_, …, *y_n_* for the *y-vector*, respectively. The preferred firing distance of each neuron *x_i_* or *y_i_* to its respective wall is denoted as *p_x_*(*i*, *l_y_*) or *p_y_*(*i*, *l_x_*). *l_x_* (respectively *l_y_*) represents a specific length to which the encoded length *p_y_*(*i*, *l_x_*) (respectively *p_x_*(*i*, *l_y_*)) will be scaled. This scaling is needed in order for the WDCs to be adaptive for environments of different sizes. For choosing *l_x_* (respectively *l_y_*), we have 2 options: (i) setting it as the sum of the distances from the agent’s current position to *w_x_* and to the opposing wall of *w_x_* (respectively of *w_y_*) $\bar{w_{x}}$, or (ii) as $l_{x}^{\max}≔d(w_{x},\bar{w_{x}})$ $\left(\text{respectively}\;l_{y}^{\max}≔d\left(w_{y},\bar{w_{y}}\right)\right)$, where *d* represents the distance function and $\bar{w_{x}}$ denotes the parallel line to *w_x_* that passes through the point inside the cage farthest away from *w_x_*. In option (i), the distances encoded by the WDCs are scaled down if the walls, whose distance is being encoded, are closer to each other at the agent’s current position. This scaling is relative to the width of the cage at that particular position. Conversely, in option (ii), no scaling of WDC densities occurs throughout the cage. One approach for establishing *p_x_*(*i*, *l_y_*), is to evenly distribute the firing peaks from 0 to *l_y_* e.g.:

$$p_{x}\left(i\right)=l_{y}\cdot \frac{i}{n}$$ (2)

$$p_{y}\left(i\right)=l_{x}\cdot \frac{i}{n}$$ (3)

**Fig. 4.**
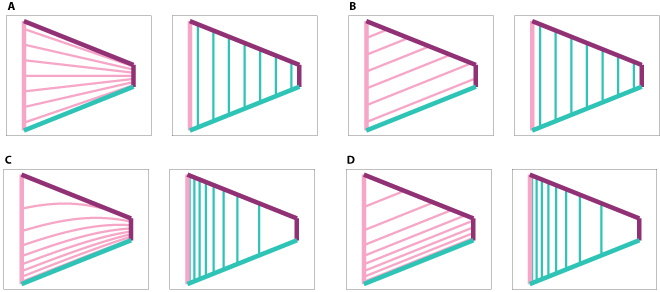
Comparison of the 2 WDC distribution options (i) (left column) and (ii) (right column), with constant (upper row) and exponentially decreasing distribution (lower row) functions *f*. Each pink line corresponds to the maximum of the firing field of a *y-vector* cell and the cage wall *w_y_* is also marked in pink. Each green line corresponds to the maximum of the firing field of an *x-vector* cell and *w_x_* is marked in green. (A) The WDC distribution follows option (i) and the distribution function *f* is constant. (B) The WDC distribution follows option (ii) and the distribution function *f* is constant. (C) The WDC distribution follows option (i) and the distribution function *f* is exponentially decreasing. (D) The WDC distribution follows option (ii) and the distribution function* f *is exponentially decreasing.

Figure 4A and B illustrate 2 examples of WDC distributions, demonstrating the distinction between options (i) and (ii) when employing this form of *p_x_*(*i*).

As it has been experimentally observed, BVCs are more densely packed if they encode shorter distances [43]. In order to incorporate these results into our model, we also choose another implementation for *p_x_*(*i*). We increase the firing peak density of distance encoding neurons *x_i_* when the distance *p_x_*(*i*) they are encoding is smaller. Based on experimental data, one can make a guess for a function *f* : [0, 1] → *ℝ*, where $f\left(x/l_{y}^{\max}\right)$ represents the preferred distance density of the *x-vector* at distance *x* from *w_y_*. In the rest of this paragraph, an approximation of *p_x_* from *f* will be derived. The objective is to derive a recursive equation for *p_x_*(*i*) that is dependent on *p_x_*(*i* − 1). If *a*(*i*) ≔ *p_x_*(*i*) − *p_x_*(*i* − 1), then *p_x_*(*i*) has to satisfy the constraint of $f\left(\left(p_{x}\left(i-1\right)+a\left(i\right)/2\right)/l_{y}^{\max}\right)=a{\left(i\right)}^{-1}$. By rearranging this equation, we obtain:

$$p_{x}\left(i\right)=f{\left(\frac{p_{x}\left(i-1\right)+a\left(i\right)/2}{l_{y}^{\max}}\right)}^{-1}+p_{x}\left(i-1\right)$$ (4)

The goal is to obtain a solution for *p_x_*(*i*) by solving Eq. 4. However, it is important to note that Eq. 4 itself is not such a solution, as the variable *p_x_*(*i*) is contained within the term *a(i)*.

Finding an explicit solution for *p_x_*(*i*) in Eq. 4 for unknown *f* is challenging due to the occurrence of *p_x_*(*i*) in the term *a*(*i*). To overcome this difficulty, an approximation *A* is employed by assuming that $f\left(\left(p_{x}\left(i-1\right)+a\left(i\right)/2\right)/l_{y}^{\max}\right)\approx f\left(p_{x}\left(i-1\right)/l_{y}^{\max}\right)$. This approximation is based on the assumption that the magnitude of *a*(*i*) is generally much smaller than *p_x_*(*i* − 1). By substituting this approximation into the previous calculation, the following expression is obtained:

$$p_{x}\left(i\right)\approx f{\left(\frac{p_{x}\left(i-1\right)}{l_{y}^{\max}}\right)}^{-1}+p_{x}\left(i-1\right)$$ (5)

Furthermore, we aim to provide flexibility in specifying the number *n* of *x-vector* neurons in our model. It is important to note that it is not possible for the densities of these neurons to be exactly equal on the critical points to the function *f* for all except at most one value of *n*. However, the densities of these neurons can be on the critical points proportional to *f* without being equal to *f* itself. To accommodate the experimenter’s ability to set the number of neurons to an arbitrary *n*, we introduce a modified function *f*′ in place of *f*. Replacing *f* with *f*′ in Eq. 4 and solving for *p_x_*(*i*) allows for customization of the number of neurons in the model.

$$f^{\prime }\left(x\right)≔f\left(x\right)\cdot \frac{n}{\int_{0}^{l_{y}}‍f\left(u/l_{y}^{\max}\right)\;du}$$ (6)

As evident in Eq. 6, the scaling of *f* varies depending on whether option (i) or option (ii) is adopted. Note that when option (i) is utilized, both *l_y_* and consequently *f*′ become dependent on the current position of the agent. Under option (i), *f*′ undergoes scaling such that each of the *n* WDCs exhibits a firing field spanning the entire width or height of the enclosure. Conversely, under option (ii), *f*′ is scaled to ensure that each of the *n* WDCs possesses a firing field with at least one point within the enclosure. Figure 4C and D illustrate 2 example WDC distributions using options (i) and (ii), when employing Eq. 5 for *p_x_*(*i*) and choosing *f*(*x*) = *c*_1_ · *e*^*c*_2_ · *x*^, were *c*_1_ and *c*_2_ are constants.

It is important to note that, when employing the approximation *A*, it is crucial to perform numerical validation to ensure that it does not introduce substantial deviations from the original solution. Let ${\hat{p}}_{x}\left(i\right)≔f^{\prime }{\left(\frac{{\hat{p}}_{x}\left(i-1\right)}{l_{y}^{\max}}\right)}^{-1}+{\hat{p}}_{x}\left(i-1\right)$, ${\hat{p}}_{x}\left(0\right)≔p_{x}\left(0\right)$ be the position of firing peak of WDC *i*, if this simplification is employed. Let $\hat{a}\left(i\right)≔{\hat{p}}_{x}\left(i\right)-{\hat{p}}_{x}\left(i-1\right)$. Now we can formulate the density of the approximated firing peaks at the points in $X:=\left\{\left.\left({\hat{p}}_{x}(i-1)+\frac{\hat{a}(i)}{2}\right)/l_{y}^{\text{max}}\right|i\in \{0,...,n-1\}\right\}$. They are $\hat{f}\left(\left({\hat{p}}_{x}\left(i-1\right)+\frac{\hat{a}\left(i\right)}{2}\right)/l_{y}^{\max}\right)≔{\left({\hat{p}}_{x}\left(i\right)-{\hat{p}}_{x}\left(i+1\right)\right)}^{-1}$. The more similar *f*′ and $\hat{f}$ are, the better the approximation worked. There are different ways to quantify their difference. We used this method:

$$\epsilon \left(l_{i}\right)≔\frac{1}{|X|}\sum_{x\in X}‍\frac{|\hat{f}\left(x\right)-f^{\prime }\left(x\right)|}{f^{\prime }\left(x\right)},\text{where}\;\;i\in \left\{x,y\right\}$$ (7)

In most experiments of this work, we use a constant *f*, where no numerical validation is needed. Just in Section “Grid distortions in nonrectangular trapezoids” in the paragraph “Nonlinear Distortion”, exponentially decreasing *f* with option (i) is employed. For that case, *ϵ* had the maximum value of 0.015 and the average value of 0.012.

Having established the preferred firing distances for both neuron vectors, we can now analytically describe the firing characteristics of the WDCs. The firing rate *x_i_* of *x-vector* neuron *i* convergences to 1 when the distance to *w_y_* is closer to the preferred distance of *x_i_* compared to the preferred distances of all other neurons in the *x-vector*. Conversely, the firing rate converges to 0 if another neuron is closer. This convergence behavior follows the convergence function described in [44]. Furthermore, the time constant *τ* is set to 50 ms, determining the convergence’s speed. Let *d*(*w_y_*, *t*) represent the distance to *w_y_* at time *t*, and let $R(x_{i})≔(p_{x}\left(i\right)-\frac{p_{x}\left(i\right)-p_{x}\left(i-1\right)}{2},p_{x}\left(i\right)+\frac{p_{x}\left(i+1\right)-p_{x}\left(i\right)}{2}]$.With these definitions, we can now provide the expression for *x_i_* as follows:

$$x_{i}\left(t+\Delta t\right)=\left\{\begin{array}{cc} \left(1-\frac{\Delta t}{\tau }\right)\cdot x_{i}\left(t\right)+\frac{\Delta t}{\tau } & \text{for}\;\;d(w_{y},t+\mathrm{\Delta t})\in R\left(x_{i}\right)\\ \left(1-\frac{\Delta t}{\tau }\right)\cdot x_{i}\left(t\right) & \text{otherwise} \end{array}\right.$$ (8)

In one simulation (see Section “Grid distortions in nonrectangular trapezoids”), the hyperparameters were adjusted, resulting in a higher density of WDCs in certain cage regions. In order to ensure that the PCs in our model (see Section “AND map (PCs)”) remained active for a sufficient duration to influence the GCs, the activation function of the WDCs required slight adjustments. In this context, WDCs were activated when the agent’s position fell within a distance of *r* from the preferred distance, thereby allowing multiple WDC cells to be simultaneously active. To be more precise, for that particular simulation, we redefined *R*(*x_i_*) ≔ [*p_x_*(*i*) − *r*, *p_x_*(*i*) + *r*].

### AND map (PCs)

We will model PCs, as justified in Section “Connectivity of these cells”, by providing them with input from WDCs (which are a simplification of BCVs and BCs). We will use an approach inspired by [36,37]. To accomplish this, we will neurally implement a logical AND operation to combine the inputs from the WDCs. We will combine the inputs from the WDCs, such that each PC corresponds to the output of the AND operation on a distinct pair of WDCs consisting of 1 neuron from the *x-vector* and 1 neuron from the *y-vector*.

**Fig. 5.**
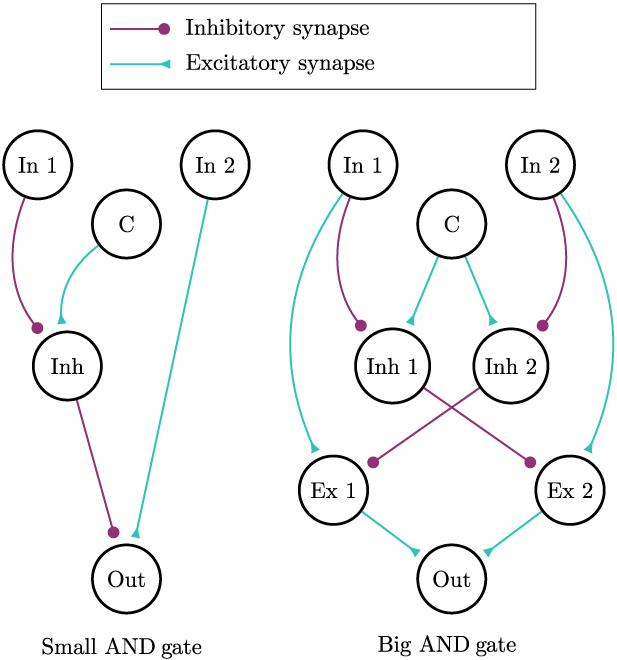
Two candidate neural circuits for implementing an AND-gate functionality are displayed. In these circuits neurons In 1 and In 2 are input neurons of the AND-gate operation. Neuron C represents a neuron with constant firing, while Out denotes the output neuron.

If *x-vector* and *y-vector* each consist of *n* neurons, then we can organize the AND-map output neurons in a *n* × *n*-matrix, which we call *A* ≕ (*a_ij_*). The desired behavior is for the output neuron *a_ij_* to be active if and only if both the *x-vector* neuron with index *i* and the *y-vector* neuron with index *j* are active. To neurally implement an AND-gate behavior, we took 2 neural circuits into consideration, which incorporate the concept of neural disinhibition. These circuits are illustrated in Fig. 5.

As evident from the comparison between the 2 circuits depicted in Fig. 5 (left and right), the circuit depicted on the left offers a simpler implementation for the AND function. This circuit exhibits computational efficiency advantages and requires a smaller number of neurons. However, it exhibits asymmetric output firing behavior when the firing rates of input neuron 1 and input neuron 2 are switched. To avoid potential side effects on the experimental results stemming from this asymmetry, we have opted for the symmetric implementation shown in Fig. 5 (right), despite its slightly reduced efficiency in the number of neurons, which could arguably also reduce the gates’ biological plausibility.

For computational efficiency, the simulations outlined in Results employed an approximation instead of a neural simulation for the neural gate. This approximation was achieved by determining the convergent value of the output for constant inputs *i*_1_ and *i*_2_. The derivation is facilitated by the property that when a neuron *A* receives a constant input sum of *x*, its firing rate converges to max(*x*, 0). This can be deduced from Eq. 1, where the input is described by the term *w* · *s* + *B* + 1. We denote *f*_∞_(*A*) =  max (*x*, 0), representing the convergent firing rate of neuron *A* when subjected to a constant input of *x*. We call the inputs to neurons “In 1” and “In 2” (see Fig. 5 (right) for a definition of the neuron names) *i*_1_ and *i*_2_. One can directly conclude *f*_∞_(In1) = *i*_1_ and *f*_∞_(In2) = *i*_2_, assuming *i*_1_ and *i*_2_ are positive. For neuron “Inh 1”, the relationship *f*_∞_(Inh1) = *f*_∞_(C) − *f*_∞_(In1) holds (with the assumption *i*_1_ ≤ 1), yielding *f*_∞_(Inh1) = 1 − *i*_1_. Following this approach, the firing rate for the Out neuron can be expressed as:

$$f_{\infty }\left(\text{Out}\right)=\frac{1}{2}\left(f_{\infty }\left(\mathrm{Ex}1\right)+f_{\infty }\left(\mathrm{Ex}2\right)\right)$$

$$\begin{array}{c} =\frac{1}{2}(\text{max(}f_{\infty }\left(\text{In}1\right)-\left(f_{\infty }\left(C\right)-f_{\infty }\left(\text{In}2\right)\right),0\text{)}\\ +\;\text{max}(f_{\infty }\left(\text{In}2\right)-\left(f_{\infty }\left(C\right)-f_{\infty }\left(\text{In}1\right)\right),0)) \end{array}$$

$$=\text{max(}f_{\infty }\left(\text{In}1\right)+f_{\infty }\left(\text{In}2\right)-f_{\infty }\left(C\right),0)$$

$$=\text{max}(i_{1}+i_{2}-1,0\text{)}$$

**Fig. 6.**
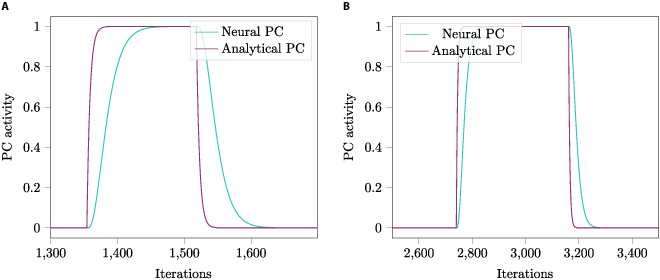
Comparison of the firing rates of a PC over time (e.g., iterations of simulation), when the approximation method outlined Section “AND map (PCs)” is used (Analytical PC) and when it is not used (Neural PC). For this comparison, a square cage with a width of 20 units was traversed. (A) shows a case of low WDC density (2 times 15 WDCs) and (B) shows a case of high WDC density (2 times 500 WDCs). For the simulation conducted for (right), the WDCs were activated by distance from the firing peak location (*r* = 1) due to their high density, as discussed at the end of Section “Wall distance cells”. Note that the PC stays active over more iterations due to this different activation method.

This simplified result accelerates the computation of the neural AND gate. However, it is important to note that this simplification assumes immediate convergence and leads to slightly different outputs of the AND gate. Figure 6 shows 2 comparisons of the activation of a PC when this approximation method is employed.

### Hebbian learning

The synaptic weights from the PCs to the GCs undergo training via Section “Hebbian learning”. We use the same Hebbian learning update rules and parameters described in [44]. Let {*g*_0_, …, *g*_*n*−1_} be the set of GCs and {*p*_0_, …, *p*_*m*−1_} denote the set of PCs. Let *s_i_* represent the current firing rate of *p_i_*, *r_j_* represent the current firing rate of *g_j_*, and *r*_max_ represent the maximum current firing rate among all GCs. First, at each time step *t*, the coactivation *α_ij_* is computed between *p_i_* and *g_j_*.

$$\alpha_{\mathrm{ij}}=s_{i}\cdot \frac{r_{j}}{r_{\max}}-\alpha_{\mathrm{th}}$$ (9)

The threshold *α_th_* = 0.05 (in [44], *α*_th_ was set to 0.05) was set to determine whether a weight update should occur. This threshold was employed to avoid a weight decay toward zero of *w*_*i*,*j*_ if *p_i_* is inactive for a while, as the agent returns to the firing location of *p_i_* only after a certain amount of time during the cage traversal. If *α_ij_* is less than zero, the corresponding weight will not be updated, as described by the following equation, which governs the temporal evolution of the weights.

$$\frac{{dw}_{\mathrm{ij}}}{dt}=\left\{\begin{array}{cc} 0, & \text{for}\;\alpha {\;}_{\mathrm{ij}}\;\leq 0\\ \frac{\alpha_{\mathrm{ij}}-w_{\mathrm{ij}}}{\tau }, & \text{for}\;\alpha {\;}_{\mathrm{ij}}\;>0 \end{array}\right.$$ (10)

If a PC *p_j_* and a GC *g_j_* often fire simultaneously, *w_ij_* will be strengthened. Following [44], we set the time constant *τ* to 10 ms. To balance the calibration input and the velocity input to the GCs, we scale each weight by a constant factor *k*. In [44], the same approach was employed with *k* = 0.05. In our case, we will use the same value for *k* as a general default. However, if the velocity transformation module (see Section “Velocity transformation module”) fails to match the calibration input, we may increase *k* to 3. We do this since if there is a systematic mismatch between path integration input and calibration input, we increase the impact of the calibration input. This is done to make the effects of calibration input on grid distortions more apparent. Consequently, the total calibration input to *g_j_* is computed as follows:

$$b_{j}=k\sum_{i=0}^{m-1}‍w_{\mathrm{ij}}\cdot s_{i}$$ (11)

Adding Eq. 11 to Eq. 1, we obtain an equation describing the spiking vector of the GC model. Let *B_c_* ≔ (*b*_0_, …, *b*_*n*−1_), *r* ≔ (*r*_0_, …, *r*_*n*−1_), *w_G_* be the weight matrix describing how the GCs are connected with each other, and *B_v_* be a vector filled with information about path integration input (more details about *w_G_* and *B_v_* can be found in [42]).

$$\tau \frac{dr}{dt}+r=\text{max}\left(w_{G}\cdot r+B_{c}+B_{v}+1,0\right)$$ (12)

**Fig. 7.**
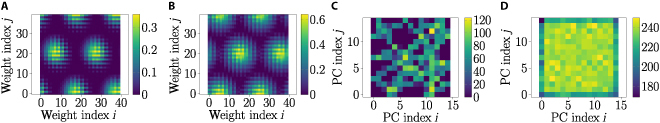
(A) and (B) illustrations present the weights of a given PC to the grid module after 5 · 10^4^ and 5 · 10^6^ iterations. The weights’ indices *i* and *j* correspond to the indices of the receiving GC (see Fig. 3 for a visual representation of GC index assignment). Panels (C) and (D) depict the sum of output weights for each PC after 5 · 10^4^ and 5 · 10^6^ iterations. The high amount of darkened PCs in panel (C) indicates that not all PCs have been trained after 5 · 10^4^ iterations. Movie S2.1, in the supplementary materials, showcases the evolution of these plots over time.

Figure 7 visualizes how weights have changed after 5 ·10^4^ and 5·10^6^ iterations of traversal in a square environment.

### Velocity transformation module

Suppose that the calibration input induces a distorted grid pattern, while the velocity input to the GCs would naturally produce an undistorted grid. In this scenario, a constant contradiction arises between velocity and the calibration input. To resolve this contradiction, we will manually transform the velocity input to align with the calibration input. This approach finds some support in experimental evidence that highlights the adaptability of speed cells to environmental changes, assuming their role in driving path integration [45]. Additionally, an experiment has likely observed a recalibration of path integration gain in response to a mismatch between intrinsic and extrinsic movement cues [46]. Furthermore, rescaling the environment in one direction led to rescaling the velocity signal in the same direction [45]. A neural circuit could replace this Section “Velocity transformation module” in future work to explore the natural mechanisms behind these effects.

#### Deriving position transformation

Before we derive a function *g* that transforms velocity in such a way, it is useful to derive a function *h* that performs the same translation but for position instead of velocity. Let the training environment be the environment in which the training has been conducted, and the test environment be the environment that the agent traverses after it has been trained. We are looking for *f*, such that if a PC *p* has its firing peak at (*x*, *y*)*^T^* in the test environment, then *h*((*x*, *y*)*^T^*) is the firing peak of *p* in the training environment. We will derive *h* for option (ii) and constant WDC distribution function *f* (see Section “Wall distance cells” for a definition of option (ii) and *f*). For the rest of this section, we further assume that the training environment is square and that in both the training and test environments *w_x_* and *w_y_* (see Section “Wall distance cells” for a definition *w_x_* and *w_y_*) intersect at (0, 0) and that for both environments, the environment lies at counterclockwise direction of *w_x_* and clockwise direction of *w_y_*. Let *φ* be the angle between *w_x_* in the test environment and the x-axis. Let *ψ* be the angle between *w_y_* in the test environment and the x-axis. Let $l_{x}^{a}$ be defined as $l_{x}^{\max}$ for the training environment (see Section “Wall distance cells” for the definition of $l_{x}^{\max}$), and let $l_{x}^{b}$ be defined as $l_{x}^{\max}$ for the test environment. We define $l_{y}^{a}$ and $l_{y}^{b}$ analogously. Let $s_{x}≔l_{x}^{a}/l_{x}^{b}$ and $s_{y}≔l_{y}^{a}/l_{y}^{b}$. *s_x_* represents the scale factor that describes the difference of the distances of *w_x_* and $\bar{w_{x}}$ in the training and test environment. As each PC is uniquely identified from the pair of WDCs that activates it, we can conclude from Eq. 2 that *h*((*x*, *y*)*^T^*) = (*d_x_* · *s_x_*, *d_y_* · *s_y_*)*^T^*. Everything left is to derive an expression for *d_x_* and *d_y_*. One can use that given

$$\left(\begin{array}{c} x\\ y\\ z \end{array}\right)\times \left(\begin{array}{c} \cos\;\alpha \\ \sin\;\alpha \\ 0 \end{array}\right)=\left(\begin{array}{c} 0\\ 0\\ d \end{array}\right)$$ (13)

, ∣*d*∣ represents the distance from the line, which goes through (0, 0) and has angle *α* to the x-axis, to the point (*x*, *y*). The symbol × denotes the cross product. If (*x*, *y*) is counterclockwise of this line, *d* will be negative and otherwise positive. This gives expressions for *d_x_* and *d_y_*:

$$\begin{array}{c} \left(\begin{array}{c} x\\ y\\ z \end{array}\right)\times \left(\begin{array}{c} \cos\;\varphi \\ \sin\;\varphi \\ 0 \end{array}\right)=\left(\begin{array}{c} 0\\ 0\\ -d_{y} \end{array}\right)\;\text{and}\;\left(\begin{array}{c} x\\ y\\ z \end{array}\right)\times \left(\begin{array}{c} \cos\;\psi \\ \sin\;\psi \\ 0 \end{array}\right)=\left(\begin{array}{c} 0\\ 0\\ d_{x} \end{array}\right) \end{array}$$ (14)

Note that we wrote −*d_y_* since we assumed earlier that the cage is counterclockwise of *w_x_*. The 2 equations can be simplified to a 1-dimensional linear equation. This allows us to express *h* as a linear transformation:

$$D≔\left(\begin{array}{cc} s_{x}\text{sin}\;\psi & -s_{x}\;\text{cos}\;\psi \\ -s_{y}\;\text{sin}\;\varphi & s_{y}\;\text{cos}\;\varphi \end{array}\right)$$ (15)

$$h\left(\left(\begin{array}{l} x\\ y \end{array}\right)\right)=\left(\begin{array}{l} d_{x}s_{x}\\ d_{y}s_{y} \end{array}\right)=D\cdot \left(\begin{array}{l} x\\ y \end{array}\right)$$ (16)

#### Deriving velocity transformation

Let *g* : *ℝ*^2^ → *ℝ*^2^ denote the function that transforms a velocity vector, such that the resulting vector creates path integration input that does not contradict the calibration input. We will derive an algorithmic expression for *g*: Let (*v_x_*, *v_y_*)*^T^* be a velocity vector for the time frame Δ*t*. This means that if, given the agent was standing at time *t* at position (*b_x_*, *b_y_*)*^T^*, at time *t* + Δ*t*, the agent was standing at position ${\left(b_{x}^{*},b_{y}^{*}\right)}^{T}\;:=\;{\left(b_{x}+v_{x},b_{y}+v_{y}\right)}^{T}$. We define *p*_0_ to be the PC with the firing peak of (*b_x_*, *b_y_*)*^T^* in the test environment and *p*_1_ to be the PC with the firing peak of ${\left(b_{x}^{*},b_{y}^{*}\right)}^{T}$ in the test environment. We define (*a_x_*, *a_y_*)*^T^* to be the firing peak of *p*_0_ in the training environment, and we define ${\left(a_{x}^{*},a_{y}^{*}\right)}^{T}$ to be the firing peak pf *p*_1_ in the training environment. We define ${\left(v_{x}^{a},v_{y}^{a}\right)}^{T}:=\;{\left(a_{x}^{*},a_{y}^{*}\right)}^{T}-{\left(a_{x},a_{y}\right)}^{T}$. Now *g* would remove the contradiction between PC firing and velocity input if *g*((*v_x_*, *v_y_*)*^T^*) was mapped to ${\left(v_{x}^{a},v_{y}^{a}\right)}^{T}$, since the way *g* creates the velocity input, that was present in the training cage, given the PC firing. Thus we obtain ${\left(v_{x}^{a},v_{y}^{a}\right)}^{T}=\;g({\left(v_{x},v_{y}\right)}^{T})$. It is important to note that, from the way we constructed *g*((*v_x_*, *v_y_*)*^T^*), *g*((*v_x_*, *v_y_*)*^T^*) is not necessarily independent of the current position of the agent (*b_x_*, *b_y_*)*^T^* given (*v_x_*, *v_y_*)*^T^*. As we will see, by applying the same assumptions as in Section “Deriving position transformation”, the translated velocity vector will be independent of the agent’s current position. These assumptions are that option (ii) and a constant WDC distribution function *f* are employed (see Section “Wall distance cells” for definitions of option (ii) and *f*). For the rest of this section, we further assume that the training environment is a square, that both in the training as well as in the test environment *w_x_* and *w_y_* (see Section “Wall distance cells” for a definition *w_x_* and *w_y_*) intersect at (0, 0) and that for both environments, the environment lies counterclockwise of *w_x_* and clockwise of *w_y_*. Using these assumptions, we can utilize the terms of *h* and *D* derived in Section “Deriving position transformation”, which describe position transformation. Namely *h*((*x*, *y*)*^T^*) = *D* · (*x*, *y*)*^T^* was derived, such that *h*((*b_x_*, *b_y_*)*^T^*) = (*a_x_*, *a_y_*)*^T^*. From *h*, we can derive an expression for *g*.

$$\begin{array}{c} g\left(\left(\begin{array}{c} v_{x}\\ v_{y} \end{array}\right)\right)=\left(\begin{array}{l} v_{x}^{a}\\ v_{y}^{a} \end{array}\right)=\left(\begin{array}{l} a_{x}^{*}\\ a_{y}^{*} \end{array}\right)-\left(\begin{array}{l} a_{x}\\ a_{y} \end{array}\right)=h\left(\left(\begin{array}{l} b_{x}^{*}\\ b_{y}^{*} \end{array}\right)\right)\\ -h\left(\left(\begin{array}{l} b_{x}\\ b_{y} \end{array}\right)\right)=D\cdot \left(\left(\begin{array}{l} b_{x}^{*}\\ b_{y}^{*} \end{array}\right)-\left(\begin{array}{l} b_{x}\\ b_{y} \end{array}\right)\right)=D\cdot \left(\begin{array}{l} v_{x}\\ v_{y} \end{array}\right) \end{array}$$ (17)

As it becomes visible, in this case, *g* is independent of the current position and *g* = *h*. This term for *g* was also empirically validated by examining whether the transformed velocity vector accurately predicted the positional offsets of a pair of PCs.

For the case where option (i) (defined in Section “Wall distance cells”) or no constant distribution function *f* is used, deriving the function *g* becomes mathematically challenging. The resulting translation from velocity in the experimenter’s coordinates to velocity in the agent’s coordinates is dependent on the agent’s current position. However, to evaluate the grid distortion produced by our calibration mechanism, it is sufficient to examine the calibration input in distorting environments. To achieve this in simulations where the WDCs are not evenly distributed, we increase the value of *k* (*k* is defined in Section “Hebbian learning”) to a sufficiently large magnitude within the interval [1, 3], ensuring that the calibration input exerts a stronger influence on the GCs compared to the slightly mismatched velocity input.

## Results

### Proof of stability

**Fig. 8.**
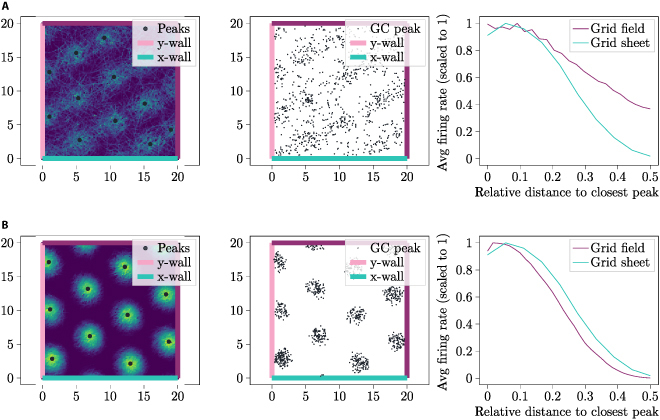
This figure presents a comparison of the field states between the uncalibrated and the calibrated GC fields after 5 · 10^6^ simulation iterations. In panel (A), calibration is off, and in panel (B), calibration is on. Panels (A) (left and middle) and (B) (left and middle) display the average firing distribution over space of a GC. Panels (A) (right) and (B) (right) depict the profiles (as defined in Section “Proof of stability”) of the field of the GC and of the grid sheet for the cases of calibration and no calibration, respectively. The black dots in (A) (middle) and (B) (middle) are each 1,000 uniform randomly chosen locations, where the GC fired higher than the 90th quantile of grid firing. Movie S1.1 in the supplementary materials corresponds to (A) and Movie S1.2 to (B).

Before examining the grid distortions produced by our model, it is useful to verify the effectiveness of our calibration circuit in stabilizing the grid. This stabilization ensures the GC fields remain stable, preventing error accumulation during extended periods of path integration. To validate this, we simulated the GC network, receiving intrinsic motion inputs from a virtual Pybullet robot, without calibration for 5 · 10^6^ time steps, where each time step Δ*t* was set to Δ*t* = 10^−2^ s. Then, we conducted simulations of the model for the same amount of time steps and same value of Δ*t*, providing it with calibration input from an untrained calibration module while simultaneously training this module. We deployed constant *f* (see Section “Wall distance cells”) and used 15 WDCs for each coordinate axis. In Fig. 8, the time-accumulated output of a single GC is depicted for both scenarios. In the supplementary materials, Movies S1.1 and S1.2 illustrate a GC’s field’s temporal progression and input. These movies showcase the evolution over time in both the noncalibration (Movie S1.1) and the calibration simulation (Movie S1.2). Movie S1.1 depicts the state of different subnetworks over time. A comparison of the fields in Fig. 8 observed in the absence of calibration and in the presence of calibration reveals differences. Specifically, in the no-calibration scenario, the field appears less defined and exhibits a more scattered distribution of firing values compared to the calibration scenario.

To quantitatively measure the stability of the GC network, we conducted a comparison between the firing distribution of a grid field (see Fig. 8B) and the firing distribution of a grid sheet. The grid sheet represents the arrangement of all GCs of the CAN by their indexes (see Fig. 3), serving as the reference for a perfect grid field.

Therefore, we created for each of the 2 datasets a curve that depicts the average firing strength as a function of its relative distance (We define the term “relative distance” as the absolute distance divided by the average absolute distance between neighboring firing peaks. Within the grid field, the absolute distance between 2 points is determined by their Cartesian coordinates, measured using the Euclidean distance. Conversely, in the grid sheet, the absolute distance between 2 neurons is defined as the Euclidean distance between their respective index tuples. For more detailed information on the indexing of GCs, refer to [41].) to the nearest peak. These curves, referred to as profiles, are illustrated in Fig. 8B (right) and A (right). The final grid sheet profile was obtained by averaging the grid sheet profiles across numerous simulation steps.

**Fig. 9.**
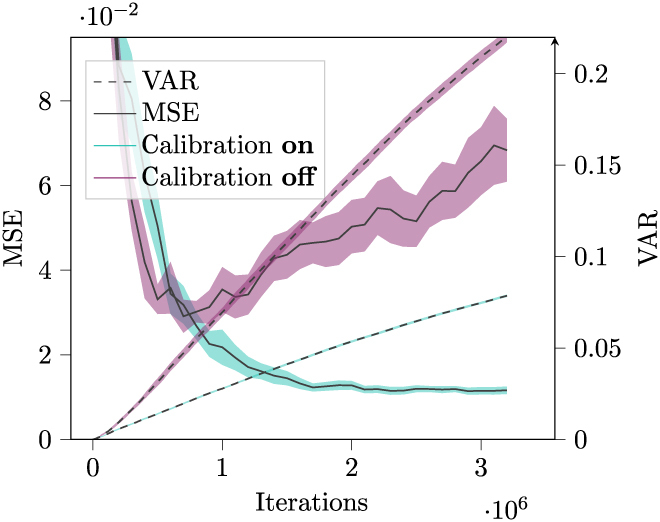
This figure illustrates the MSE values between the grid sheet and the grid field profiles and the VAR values over simulation time (see Section “Proof of stability” for an explanation of MSE and VAR). Both the means and 90% confidence intervals (assuming normally distributed deviation from the mean) of the MSE and VAR were estimated using 20 simulations per sample (20 simulations with calibration turned on and 20 with calibration turned off) for the MSE and 16 simulations per sample for the VAR. The turquoise lines represent the MSE (solid line) and VAR (dashed line) developments when calibration was enabled, while the purple lines represent the scenario where calibration was disabled. During the early stages of the cage traversal (∼5 · 10^5^ first iterations), insufficient data are available to entirely compute the grid field. The first 2 s of Movie S1.2 (top, left) showcase this incomplete grid field. This leads to an incomplete computed grid field profile as seen in the first 2 s of Movie S1.2 (top, third from left). The incomplete grid field in the early stages of the simulation results in a very high MSE that does not adequately reflect the grid’s stability during this stage.

In the final step of the analysis, the mean square error (MSE) between the grid field and the grid sheet profiles was computed. A lower MSE indicates a higher resemblance between the two, indicating higher stability of the GC network. Figure 9 displays the progression of the MSE in both the calibration and the no-calibration scenario over the course of 3.2 · 10^6^ simulation steps.

In the scenario without calibration, after 20 simulations, the MSE reached a mean value of 68.3 · 10^−3^ (±7.42 · 10^−3^, 90% confidence interval, assuming normally distributed error). In the scenario with calibration, after 20 simulations, the MSE reached a mean value of 11.6 · 10^−3^ (±0.88 · 10^−3^, 90% confidence interval, assuming normally distributed error). Thus, in the scenario without calibration, the mean obtained MSE is 5.9 times higher than in the scenario with calibration. These findings suggest that the calibration circuit may contribute positively to improving the stability of the GCs.

To gain more comparative insights, we incorporated an additional measure for assessing the stability of the GCs. This measure is the mean over the environment of the empirical variance of a GC firing at the given location. We will refer to this measure as VAR. A lower value of VAR means a higher consistency of the GC regarding its firing value and space. To approximate VAR, we partitioned the environment into 200 by 200 segments, which are denoted as *S* ≔ {*s*_*i*, *j*_| *i*, *j* ∈ {0, 1, …, 199}}. During the simulation, we added the current firing rate *r*(*t*) of a GC each time it encountered a segment *s*_*i*,*j*_ ∈ *S* to the set *R*_*i*,*j*_. Subsequently, for each segment *s*_*i*,*j*_, we calculated the empirical variance *V*_*i*,*j*_ of the GC activity at this segment $V_{i,j}≔\frac{1}{|R_{i,j}|}\sum_{r\in R_{i,j}}‍{\left(r-\bar{r}\right)}^{2}$, where $\bar{r}≔\frac{1}{|R_{i,j}|}\sum_{r\in R_{i,j}}‍r$. Then, we obtained this term for VAR:

$$\mathrm{VAR}≔\frac{1}{|S|}\sum_{s_{i,j}\in S}‍V_{i,j}$$ (18)

The mean time evolution of VAR for both the calibration and the noncalibration scenarios is depicted in Fig. 8. In the scenario without calibration, after 16 simulations, the VAR reached a mean value of 0.22 (±3.4 · 10^−3^, 90% confidence interval, assuming normally distributed error). In the scenario with calibration, after 16 simulations, the MSE reached a mean value of 0.079 (±0.64 · 10^−3^, 90% confidence interval, assuming normally distributed error). Thus, the mean VAR obtained in the scenario without calibration is 2.8, which is times higher than in the scenario with calibration.

### Reproducing grid distortions

This section aims to investigate the ability of the model to reproduce different grid distortions. The simulations conducted in this study follow a consistent procedure: the model is initially trained in a square environment (also referred to as the training environment), and subsequently, the agent navigates through environments of various arbitrary shapes (also referred to as test environments).

For all simulations except the simulation in the paragraph “Nonlinear Distortion” in Section “GC and PC distortions”, option (ii) and a constant distribution function *f* were employed (see Section “Wall distance cells” for definitions of *f* and option (ii)). Thus, for these simulations, both in the training and test environments, *k* could be set to 0.05 (see Section “Hebbian learning” for a definition of *k*).

For the test environment in the paragraph titled “Nonlinear Distortion” in Section “GC and PC distortions”, a value of 3 was assigned to the parameter *k*. This value of *k* was necessary, as in that simulation option (ii) and a nonconstant distribution function *f* were employed. This setting causes the velocity transformation module to be unable to match the grid distortion, as discussed in Section “Velocity transformation module”. This choice of *k* in the test environment ensures that the grid output is predominantly determined by the calibration input, which is the area of interest when exploring the grid distortions of this model.

#### Grid distortions in nonsquare rectangles

In Section “GC and PC distortions”, we examined the experiment conducted in [12], where rats were familiarized with a square cage, which was then scaled down along the x- or y-axis. This scaling directly affected the resulting grid of the rats’ GCs, causing it to also be scaled down along the x-axis (analogously, for the y-axis case).

**Fig. 10.**
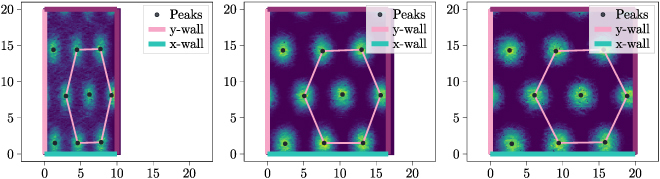
From left to right, the displayed images show the field of a GC after 3.2 · 10^6^ iterations in rectangular environments of varying thickness, where the calibration circuit used in each environment was trained within a square enclosure. As the enclosure is scaled down along the x-axis, the grid is also scaled down along the x-axis. (Left) MSE: 2.9 · 10^−2^, VAR: 0.29; (middle) MSE: 0.22 · 10^−2^, VAR: 0.096; (right) MSE: 0.83 · 10^−2^, VAR: 0.070. Note that the MSE and VAR (explained in Section “Proof of stability”) values only reflect the values for these specific fields and are not averages over multiple trials as in Fig. 9 and thus should not be generalized.

To replicate this experiment, we trained the calibration circuit within a square enclosure and subsequently evaluated how the grid would form within different rectangular environments. In each environment, we deployed constant *f* (see Section “Wall distance cells”) and used 15 WDCs for each coordinate axis. Figure 10 compares the grid in the trained environment and the grid within various rectangular environments. Consistent with the findings reported in [12], our model demonstrates a similar pattern where the resulting grid is scaled down along the same axis as the square cage was scaled down to form the rectangle.

#### Grid distortions in nonrectangular trapezoids

In Section “GC and PC distortions”, we discussed the research conducted in [11], which investigated the behavior of GCs within trapezoidal enclosures. Their study also revealed that a fraction of grid modules only exhibited a very small or negative increase in ellipticity, when transitioning into the tetragonal environment, while others displayed a greater ellipticity. Moreover, considerable variance was observed in changes in the grid’s wavelength and orientation across the trapezoidal environment, suggesting the possibility of some grid modules only experiencing orientation changes as low as in the square environment. Possibly consistent with these findings, our analysis identified a parameter setting that induced an increase in ellipticity without altering orientation or wavelength throughout the trapezoid (refer to the paragraph “Linear Distortion”). Additionally, another parameter setting reproduced changes in orientation (although to a lesser extent) and wavelength across the trapezoid, while maintaining constant ellipticity (refer to the paragraph “Linear Distortion”). Notably, our investigation did not identify a parameter setting capable of replicating all 3 phenomena simultaneously. The potential discovery of a negative correlation between changes in ellipticity and changes in orientation or wavelength would align with the predictions of our model. If this negative correlation is not observed, the concurrent modeling of all 3 distortions could potentially be attributed to an alternative method of distributing the WDCs in future work.

In both simulations, the network underwent training within a square environment (training environment) before traversing the trapezoidal environment (test environment).

*Linear Distortion*

**Fig. 11.**
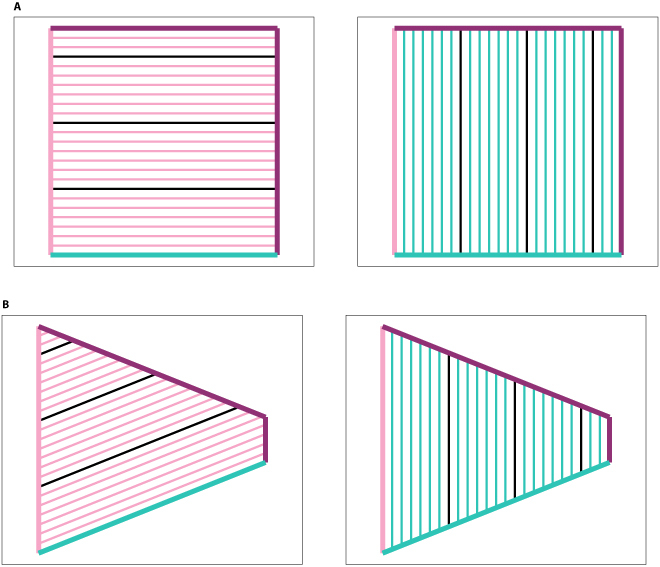
The depicted WDC distributions represent the configuration used to replicate the linear distortion. For experimental and simulation results illustrating the corresponding linear distortion, please refer to Fig. 12. In this distribution, the density of WDCs remains constant across space, and the WDC distribution follows option (ii). Every seventh peak was highlighted in black to improve visibility. (A) WDC firing peaks of *x-vector* (right) and *y-vector* (left) in the training environment. (B) WDC firing peaks of *x-vector* (right) and *y-vector* (left) in the test environment.

We ensure an even distribution of WDCs throughout space to replicate the linear distortion. This was accomplished by employing option (i) and a constant function for *f* (see Section “Wall distance cells” for further details). Twenty-five WDCs were employed for each axis, and *k* was set to 0.05. Please refer to Fig. 11 for a visual representation of this distribution.

**Fig. 12.**
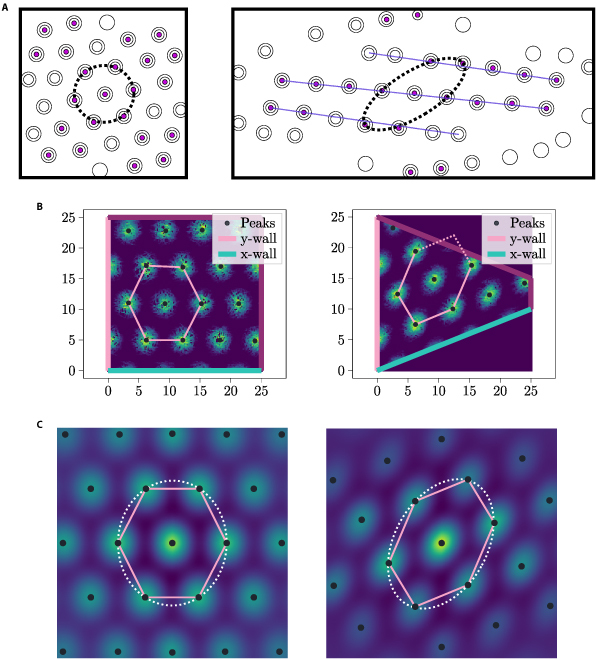
Panel (A) presents a visualization of a result from the study [11]. This result resembles the autocorrelograms of the firing fields of exemplary GCs in a square environment (left) and in a trapezoidal environment (right), demonstrating the linear transformation of the grid pattern in a trapezoidal environment. Panels (B) and (C) show the simulation results trying to reproduce the experimental observation of (A). In (B) (right), we see the grid field of a simulated GC in the trapezoidal test environment, while (B) (left) displays the output of the GC in the square training environment. Refer to Fig. 11, to see the WDC distributions utilized for this simulation. Panel (C) (left) shows the autocorelogram of (B) (left) and (C) (right) the autocorelogram of (B) (right). Ellipticity of left ellipse: 0.493, ellipticity of right ellipse: 0.746.

Figure 12 compares the simulation output with the experimental results from [11], for the WDC set aiming to reproduce the linear distortion. Both in the simulation and in the experiment from [11], the linear transformation seems to be present. Specifically, in the test environment, the grid ellipticity was 0.746, whereas in the training environment, the grid ellipticity was 0.493.

The grid ellipticity serves as a measure of the regularity of the hexagonal grid, where an ellipticity value of 0 indicates perfectly regular hexagons and an ellipticity of 1 indicates a linear transformation with a determinant of 0. The calculation of the grid ellipticity followed the methodology outlined in [11], which involved obtaining the autocorrelogram of the grid, fitting an ellipse to the central hexagon, and subsequently calculating the ellipticity of this ellipse.

*Nonlinear Distortion*

In order to reproduce the nonlinear distortions, we adopted a distribution of WDCs with an exponentially decreasing function for *f* and utilized option (ii) (see Fig. 14). A total of 130 WDCs were used for each axis, and function *f* was designed with the properties *f*(0) = 1 and *f*(0.5) = 0.1:

$$f\left(x\right)=\exp\left(x2\ln\left(0.1\right)\right)$$ (19)

Due to the high density of WDCs, for this simulation, the alternative activation rule for WDCs, as discussed at the end of Section “Wall distance cells”, was employed for this experiment with *r* = 1. Furthermore, *k* was set to 3 in the test environment since, in this scenario, the Section “Velocity transformation module” cannot account for the created distortion.

Movies S2.2.1 and S2.2.2 showcase the state of different subnetworks over time for the training phase in the square environment and for the test phase in the tetragonal environment, respectively.

In the test environment, the grid exhibited an ellipticity of approximately 0.511; in the training environment, the ellipticity was around 0.498. Unlike in the simulation, where parameters were set to reproduce the linear distortion, only a marginal increase in ellipticity was observed for this particular simulation. In [11], grid modules were observed only displaying a very small to no increase of ellipticity upon entering the trapezoidal environment, up until grid modules displayed higher increments of ellipticity.

**Fig. 13.**
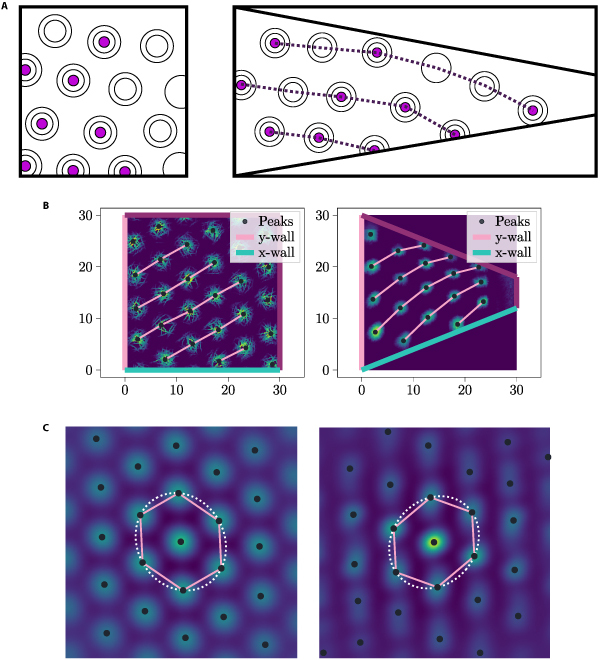
Panel (A) presents a visualization of a result from the study [11]. This particular result portrays the firing field of a sample GC, highlighting the observed changes in grid orientation throughout the trapezoidal environment (A) (right) from left to right (refer to Section “GC and PC distortions”). In (B), we present the simulation results obtained by setting firing peaks of the WDCs as depicted in Fig. 14, aiming to replicate the observed changes in orientation. (B) (left) shows the firing field of a GC in the training environment and (B) (right) in the test environment. The pink lines in (B) (left and right) indicate the nonlinear effect of the distortion and a change in orientation of the hexagonal axis corresponding to the pink lines throughout the enclosure from left to right. The vertical hexagonal axis remains, not in accordance with a change in orientation of the total grid in the experimental result from (A), constant throughout the trapezoid. Panel (C) shows autocorrelograms of simulation results in (B). (C) (left) depicts the autocorrelogram of the GC field in (B) (left) and (C) (right) the autocorrelogram of the GC field in (B) (right). Ellipticity of left ellipse: 0.498, ellipticity of right ellipse: 0.511.

In Fig. 13B, one can observe that one of the 3 grid axes exhibited a change in orientation across the trapezoid enclosure, while the other 2 axes maintained a constant orientation. This change in orientation is visually depicted by the pink curved lines in this figure. To quantitatively compare the change in orientation to the results in [11], we calculated the total difference in orientation between the left and right halves of the enclosure using the same method as described in [11]. For the left half interval ([0,15.9]) and the right half interval ([10.5,25.5]), ensuring that each half contains at least one complete hexagon, we obtained a difference of 1.6° in the trapezoid (in the trapezoid, a change in orientation of 3.39° was observed when using the left half interval of [0, 15] and the right half interval of [15, 30]). In the square, with the left interval being [0, 15] and the right interval being [15, 30], the change in orientation was 1.0°. Hence, the shift in orientation within the trapezoidal environment was 1.6 times greater than that observed in the square environment. It is important to note that the magnitudes of the orientation differences, both in the training environment and the test environment, were much smaller than the orientation changes reported in [11]. When comparing the orientations of the upper and lower halves of the environments, with the upper interval being [30,13.5] and the lower interval being [18.6,0], we obtained a difference of 6.4° in the test environment. In the training environment, with the upper interval as [30, 15] and the lower interval as [15, 0], the difference was 0.95° (in the test environment, a change in orientation of 8.8° was observed when using the lower half interval of [0, 15] and the upper half interval of [15, 30]).

**Fig. 14.**
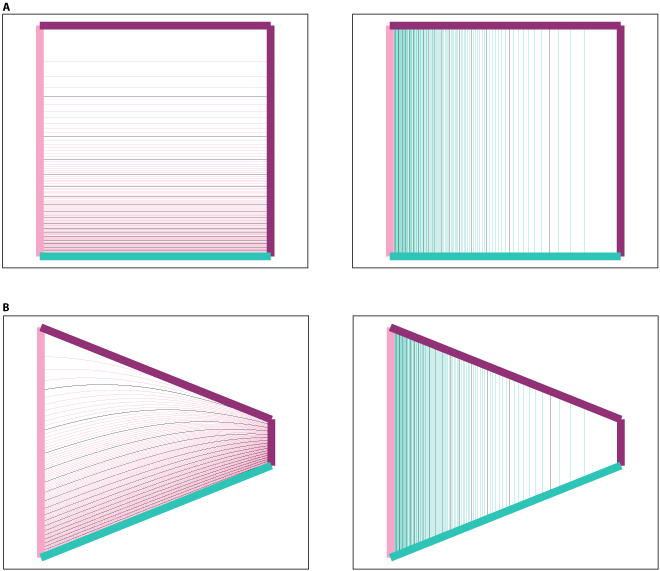
The depicted WDC distributions represent the configuration used to replicate the distortion of a change in orientation of the grid throughout the cage. To illustrate corresponding experimental and simulation results, refer to Fig. 13. In this distribution, the density of WDCs is exponentially decreasing and the WDC distribution is of option (i). Every seventh peak was highlighted in black to improve visibility. (A) WDC firing peaks of *x-vector* (right) and *y-vector* (left) in the training environment. (B) WDC firing peaks of *x-vector* (right) and *y-vector* (left) in the test environment.

The determination of grid compression or expansion relies on the analysis of the wavelength exhibited by the grid pattern. In [11] also, a change in the wavelength of the grid across the enclosure was observed. The change in wavelength of the grid can be used to identify the compression or expansion of the grid. In order to determine the wavelengths of a grid, we calculated the 2-dimensional discrete Fourier transform of the autocorrelogram of the grid field. Let *P*(*f_x_*, *f_y_*) represent the value of the Fourier transform for frequency *f_x_* in the x-direction and *f_y_* in the y-direction. We denote the set of (*f_x_*, *f_y_*) vectors for which *P*(*f_x_*, *f_y_*) is computed as *F*, which is a square grid of vectors. From *P*, we can deduce the contribution of the harmonic signal with frequency (*f_x_*, *f_y_*) to the autocorrelogram with respect to the signal’s amplitude. The normalized contribution is ∣*P*(*f_x_*, *f_y_*)∣ /*S_P_*, where *S_P_* ≔ ∑_(*f_x_*, *f_y_*) ∈ *F*_ ‍  ∣ *P*(*f_x_*, *f_y_*)∣. By treating the normalized contribution as a probability density function, for the random frequency vector (*F_x_*, *F_y_*), we can obtain a numerical approximation for the mean of the wavelength $E\left[W\right]$, where $W≔{\left(F_{x}^{2}+F_{y}^{2}\right)}^{-2}$. We obtain this term, by applying the law of the unconscious statistician (to correctly apply this law, it is important to note that the values in *F* form a square grid):

$$E\left[W\right]=\frac{\sum_{\left(f_{x},f_{y}\right)\in F}‍|P(f_{x},f_{y})|\cdot {\left(f_{x}^{2}+f_{y}^{2}\right)}^{-2}\;}{S_{P}}$$ (20)

In the training environment, we obtained a wavelength of 3.25 in the left half and 3.38 in the right half. Conversely, in the test environment, we obtained a wavelength of 4.00 in the left half and 3.77 in the right half. It is important to note that the same division in the left and right half as that used for the orientation analysis was employed in both cases (when using a left half interval of [0, 15] and a right half interval of [15, 30], wavelengths of 3.80 [left half] and 3.77 [right half] were observed in the test environment). This corresponds to a change in the wavelength by 0.23 within the trapezoidal environment and of 0.13 within the square environment.

Another measure that aids in determining a compression or expansion of the grid is the average peak distance, specifically referring to the average distance between each peak and its nearest neighboring peak. In the training environment, we obtained an average peak distance of 5.28 in the left half and 5.76 in the right half. Conversely, in the test environment, the average peak distance was 5.27 in the left half and 4.07 in the right half. It is important to note that the same spacing as that used for the orientation analysis was employed in both cases (when using a left half interval of [0, 15] and a right half interval of [15, 30], average peak distances of 5.27 [left half] and 3.86 [right half] were observed in the test environment). This corresponds to a change in the wavelength by 1.20 within the test environment and of 0.48 within the training environment. An overview of these observations is presented in Table.

**Table.** This table shows the differences in grid properties between the left and right halves of the firing fields in the square or trapezoidal environment from the experiment aiming to reproduce nonlinear distortions. The experiment is described in Section “Grid distortions in nonrectangular trapezoids”, and the firing fields of the simulated GC used for this comparison are depicted in Fig. 13. Refer to Section “Grid distortions in nonrectangular trapezoids” to see how the values in the table were obtained.

	Square	Trapezoid
Rotation diff.	1. 0^∘^	1. 6^∘^
Wavelength diff.	0.13	0.23
Average peak dist. diff.	0.48	1.20

### Reproducing PC distortions

**Fig. 15.**
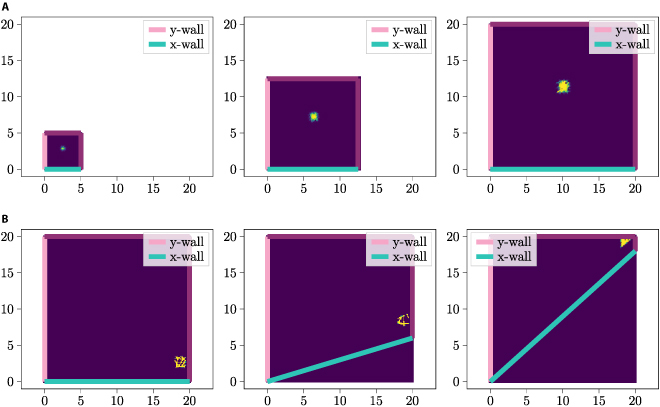
Panel (A) depicts the firing fields of a PC in rectangular enclosures of different sizes. Panel (A) (left) represents the firing field in a smaller enclosure (side length 5), (A) (middle) the firing field in an enclosure of side length 12.5. In comparison, (A) (right) shows the firing field in a larger enclosure (side length 20). It can be observed that the size of the place field increases with the size of the enclosure. Panel (B) (left) depicts the firing field of a PC of our model in a rectangular enclosure. In (B) (middle) and (right), the position of the south wall has been altered and the firing field of the same PC as in (B) (left) is depicted. The PC’s firing field exhibits movement that corresponds to the repositioning of the wall.

In Section “Connectivity of these cells”, we discussed studies that examined the relationship between place fields and environmental enclosures. It has been observed that when the size of an environment is scaled up, a proportion of the place fields also scale up [14]. We tested whether the PCs in our model exhibit similar behavior. Figure 15A shows the place field of a PC in a rectangular enclosure with a side length of 10 and the place field of the same cell in a rectangular enclosure with a side length of 20. The figure demonstrates that the size of the place field in our model has also scaled up in the larger square, which can be attributed to the increased distance between WDC firing fields.

It has been observed in [13] that certain place fields exhibited movement in relation to the cage wall, whose position underwent slight changes over multiple days. Figure 15B illustrates the firing field of a selected PC from our model in different environments characterized by varying positions of the wall *w_x_*. Notably, the place field remains in close proximity to the wall, aligning with the experimental findings of [13].

## Discussion

### Limitations and future work

#### Accurateness of simulated distortions

The model was able to reproduce the PC distortions, where place fields were rescaled [14] and where place fields changed their position, after manipulating the environment [13] (see Section “Reproducing PCs distortions”). Also, the model was able to reproduce GC distortions of [12] (see Section “Grid distortions in nonsquare rectangles”) and of [11] (see Section “Grid distortions in nonrectangular trapezoids”). Regarding [11], among the various distortions presented in Section “GC and PC distortions”, there remains one distortion that could only be partially reproduced. Specifically, in Section “GC and PC distortions”, the study conducted in [11] was presented, where various kinds of distortions were observed. One of these distortions involved a change in the orientation of the grid across the trapezoidal environment. As mentioned previously in Section “Grid distortions in nonrectangular trapezoids”, when trying to replicate this phenomenon, one of the 3 grid axes of our model exhibited a change in orientation across the trapezoidal enclosure, while the other 2 axes maintained a constant orientation. Therefore, quantitatively, we observed lower changes in the orientation of the total grid in comparison to those observed in the original experiment. It is important to mention that this work focused on modeling GC and PC distortions appearing when the environment is manipulated. Therefore, it cannot reproduce grid distortions like those in [35] occurring in square environments. In that study, a shearing-induced asymmetry of the grid was found to develop when rats gained experience with a square environment. Contrarily, the calibration circuit of this work is trained inside a square environment, and for the GC model, a CAN is used that does not create any GC distortions apart from the path integration error. As a result, when exposed to square environments, the model preserves the regular grid pattern and cannot reproduce GC distortions appearing in square environments, such as those in [35]. These distortions may be attributed to an alternative mechanism that influences the firing patterns of GCs, which is not accounted for by this work’s model.

#### WDCs are a simplification

Including more biologically plausible cell types, such as BCs and BVCs, in place of WDCs is an important consideration for future work. There are numerous possibilities for making this part of the model more biologically plausible and replacing WDCs with these 2 cell types. One potential approach, similar to the work in [15], is to develop a feedforward model that incorporates BVCs and BCs as input cells. By employing correctly configured learning rules, the model’s output layer can be tailored to simulate the behavior of PCs. By doing so, a more biologically realistic alternative to the neural AND gate described in Section “AND map (PCs)” could be implemented. This approach also holds the potential to enable the encoding of distances from all 4 walls and make it even adaptable to arbitrary convex environments. This would constitute an improvement, as the current implementation only considers distances from 2 walls, further enhancing the biological plausibility of the model.

Additionally, specifically when trying to show in future work that direct connections from BVCs to GCs are unlikely, it is important to note that simplifying using WDCs instead of BVCs may yield problems. This is due to the difference in the geometric properties of the firing field of a BVC and a WDC inside a rectangular cage. As any pair of firing fields of WDCs inside a rectangular cage is either parallel or has a 90° angle, a GC-input firing field *F_W_*(*x*, *y*), which is a linear combination of firing fields of WDCs, can be expressed as *F_W_*(*x*, *y*) = *f_X_*(*x*) + *f_Y_*(*y*), where *f_X_* is independent of *y* and *f_Y_* is independent of *x*. This means that *F_W_* can be expressed so that the *x* and the *y* coordinates are separated relatively well in the expression. This property allows easy proof that a linear combination of firing fields of WDCs inside a square cage can not even form a firing field, which is slightly useful for calibrating a GC *g*. We say a firing field *F* that serves as calibration input to a GC *g* is slightly useful if there is at least one minimal regular triangle *t* containing 3 firing peaks *g*_1_, *g*_2_, *g*_3_ of *g*, such that the minimal horizontally oriented square *s* containing *t*, contains no point *p*, such that:

• *p* is not a firing peak of *g*

• *F*(*p*) ≥  min {*F*(*a*)| *a* ∈ {*g*_1_, *g*_2_, *g*_3_}}

Due to the nonexistence of this linear combination, it is impossible for a neuron model, in which a neuron’s input is a linear combination of the firing of a set of neurons, to have weight values such that WDCs provide slightly useful input to PCs. Unfortunately, in a linear combination of BVCs *F_B_*, the *x* and the *y* value can not be separated as easily as in *F_W_*. E.g. *F_B_* cannot in general be expressed as $F_{B}(x,y)=f_{X}^{*}\left(x\right)+f_{Y}^{*}(y)$. This is true, as an “L”-shaped firing field of one single BVC, which is also a linear combination of BVCs, cannot be expressed that way. Therefore one may be careful to conclude from the unlikeliness of a direct connection from WDCs to GCs the unlikeliness of a direct connection from BVCs to GCs, especially when running simulations using WDCs.

#### Adding GC feedback for PCs

In the current model, PCs fire highly accurately due to the precise firing of WDCs. Consequently, in the current model configuration, corrective input from GCs to PCs would not substantially alter the PCs’ firing, as their accuracy is already maximized. However, it is essential to recognize that the high accuracy and density of WDCs is not likely to be biologically accurate. To explore this further, future work could artificially degrade the amount and accuracy of WDCs/BVCs and thereby also reduce the precision/number and accuracy of PCs to simulate more realistic conditions. By doing so, we could also evaluate effects that arise from neuronal inputs from GCs to PCs.

Less accurate or fewer WDCs would allow a GC-to-PC connection to increase the information about the current location of PCs and, therefore, be functional. Adding this connectivity might yield a good comparison to this model and could help to evaluate the biological plausibility of a GC-to-PC connection. One problem that might arise from adding weights from GCs to PCs is that a PC and a GC could create a positive loop and stay active forever, even after the agent has moved to another position. To avoid this potential problem, a winner-takes-it-all mechanism could be added to the PCs, similar as done in [17], where a model with a GC-PC loop was implemented to explore phenomenons such as grid realignment or global PC remapping. The winner-takes-it-all mechanism could be hardcoded or achieved by adding neural circuitry for lateral inhibition. Alternatively, GCs could not be directly connected to PCs, but each GC *g_i_* could be connected to each PC *p_j_* over a Neuron *n_ij_*. Then, a BVC providing input to a PC *p*_2_, which is neighboring a PC *p*_1_, could inhibit for all *i* the neuron *n*_*i*1_, which transmits information from *g_i_* to *p*_1_. Also interesting is the speculation about the potential impacts of such a connection on GC distortions. Suppose the network’s weights are not changed in the test environment and the Section “Velocity transformation module” does not create systematic GC–PC conflicts. In that case, no other GC distortion should occur as the velocity input to GCs creates the same distortion as the PC input from WDCs/BVCs. Thus, the weights determining inputs to PCs from BVCs or the weights determining PC–GC interactions should also be plastic in the test environment to explore new possible GC distortions if the model is built to converge into a conflict-free state (which is not a necessity). If the model is extended to have plastic weights in the test environment, it could reproduce observations on how grid distortions evolve over time. One possible effect might be that the grid distortion would slowly disappear after enough exploration, and the GCs would create the regular hexagon. In [12], to prepare an experiment, rats were familiarized with a nonsquare rectangular environment by experiencing it for 20 min on 3 consecutive days each. Afterward, a regular grid in that environment was observed. In the paper, the shape of the grid before familiarization was not mentioned, as this was unrelated to their experiment. Suppose the grid is distorted in that rectangular nonsquare environment before familiarization. In that case, that phenomenon corresponds to the potential disappearance of the grid distortion after enough simulation time in the test environment of a future model.

The future extension of adding GC-to-PC feedback would be well-aligned with research discussed in Section “Connectivity of these cells” since the exact contribution of GC firing to PC firing is not entirely clear. A comparison of the effectiveness of an extension of this model wherein GCs’ firing contributes to PC firing, in replicating experimentally observed phenomena, to the effectiveness of this model, may yield insights into this discourse.

#### Improving biological plausibility of the velocity transformation module

Moreover, within our model, the Section “Velocity transformation module” was hardcoded rather than being neurally simulated. A more refined approach would involve the development of a neural network capable of learning to translate velocity input, thereby eliminating constant contradictions between path integration and calibration input. A neural implementation could yield new insights into the neural circuits involved in position coding. This approach could potentially result in speed cell behavior aligning with the findings in [45,46]. A biologically plausible method for feeding neurally represented velocity into the GC module may be used to achieve this. If we assume that the path integration input to GCs originates from head direction and speed cell signals, it would be reasonable to propose a computational model for GCs that receives input from both head direction and speed cells or from a transformed signal derived from these sources. As changes in speed cell firings relative to environmental boundaries have been observed [45], implementing a neural mechanism that constantly fine-tunes speed and head direction cell activity to reconcile any discrepancies between path integration signals and externally derived position signals may offer a viable approach.

### Suggestions of the model and of related work

#### Interaction of grid and place cells

This model provides additional support for certain concepts discussed in Section “Connectivity of these cells”. Specifically, it reinforces the notion that PCs are substantially influenced by BCs or BVCs and that PCs provide input to GCs—potentially for calibration purposes. This notion is reinforced by the argument that its use enabled the creation of a model that closely resembled experimental evidence. If, in future work, a GC-to-PC connection was implemented (see Section “Adding GC feedback for PCs”), a comparison to this works model could provide suggestions about the plausibility of this connectivity.

#### Exploring novel grid distortions in rats familiarized in nonsquare cages

In all our conducted experiments, the calibration circuit was trained using a square enclosure. This choice was motivated, in part, by the assumption that the laboratory rats involved in the grid distortion experiments were raised in cages consisting of square or at least rectangular rooms. However, it is worth considering the potential outcomes of allowing rats to be familiarized with a trapezoidal or nonrectangular cage and then manipulating that environment to be square-shaped. To explore this scenario, one could predict a potential outcome by training the calibration circuit within a trapezoidal environment and subsequently observing the grid inside a square environment.

#### Distortions of BVCs

In Section “Wall distance cells”, we propose certain hypotheses regarding the appearance of WDC fields in environments that induce grid distortions based on the experimental finding that BVCs are less dense with a higher distance from their encoding border. The shape of the WDC fields determined the resulting grid distortion. For certain hyperparameter settings, WDCs and BVCs only exhibit the same firing field characteristics in rectangular environments, but not in nonrectangular trapezoidal environments, if we use the current behavioral definition of BVCs.

Consequently, it is plausible that a subset of BVCs within a trapezoidal environment may not exhibit the firing field described by the current behavioral definition of BVCs, as instead, this model predicts for some BVCs to act similarly as calculated in Section “Wall distance cells”, with their firing field bending along the enclosure and not being perfectly parallel toward this wall, as the conventional model predicts.

Figure 1 presents a visual comparison between the conventional BVC model and our models’ proposal for a BVC model within a trapezoidal environment, showcasing how the conventional model yields lower entropy in the BVC firing patterns compared to the newer version.

#### Possibility of different types of GCs

In Section “Grid distortions in nonrectangular trapezoids”, 2 distinct distributions of WDCs were employed to replicate 2 different experimental observations. In one case, the distribution of WDCs was set to reproduce the shear distortion that increases the ellipticity of the resulting grid. In the other case, the distribution of WDCs aimed to reproduce the change in orientation throughout the trapezoid. The ellipticity ratio of the hexagon in the resulting autocorrelogram indicated that, unlike in the first case, the increase in ellipticity observed in the second case was not substantially larger. This aligns with the high variability in ellipticity observed among different grid modules in [11], such that a fraction of GC modules exhibited almost no linear distortion. These findings give the impression that grid modules may receive inputs as a linear combination of 2 distinct input types: one that induces shear distortion in GCs and another that promotes a more pronounced change in orientation across the grid. Therefore, if the experiment conducted in [11] indeed demonstrates a correlation between a low ellipticity of a grid module and a high change in orientation of the grid throughout the trapezoid, it aligns with the predictions of our model and supports the concept of 2 types of inputs to GCs.

These 2 different types of input could potentially be attributed to a behavioral segregation of BVCs. It is plausible that certain BVCs exhibit a higher resemblance to the characteristics described in option (i), while others align more closely with those described in option (ii). By applying appropriate learning rules, it may be possible to achieve that certain PCs receive input to a greater extent from option (i) WDCs, while others receive input to a greater extent from (ii) WDCs. This would result in variations in the behavior of individual PCs, which in turn could lead to distinct distortions in different grid modules.

### Conclusion

In this study, we have developed a GC calibration model oriented on the established evidence regarding the connectivity patterns of functional navigational cell types in the hippocampal formation, as discussed in Section “Connectivity of these cells”. The primary objective of this model is to elucidate the mechanism underlying grid distortions and provide a comprehensive explanation for these phenomena. Through our modeling efforts, we could reproduce most of the experimental findings reported in [11–14]. However, it is important to acknowledge that one specific case in [11] was not entirely replicated as discussed in Section “Grid distortions in nonrectangular trapezoids”.

The present study acknowledges the notable contributions made by previous models in successfully reproducing grid distortion phenomena, as exemplified in [47,48] and [15], advancing our understanding of GCs. However, it is worth noting that these earlier models did not specifically focus on addressing the distortions observed in PCs as described in [13,14] or in cell types other than GCs. To the best of our knowledge, this study’s model is the first to comprehensively address grid distortions, PC distortions, and indirectly speed cell distortions, offering an additional perspective on the underlying mechanisms.

## Data Availability

The Git repository, which includes all the code used to generate simulation data and analyze it, can be accessed via this link: https://github.com/strammermax27/modeling_grid_distortions.
